# A model identifying characteristics predictive of successful pelvic floor muscle training outcomes among women with stress urinary incontinence

**DOI:** 10.1007/s00192-020-04583-z

**Published:** 2020-11-25

**Authors:** Kaylee C. L. Brooks, Kevin Varette, Marie-Andrée Harvey, Magali Robert, Robert J. Brison, Andrew Day, Kevin Baker, Vincent Della Zazzera, Eric Sauerbrei, Linda McLean

**Affiliations:** 1 School of Rehabilitation Sciences, University of Ottawa, Rm E260C, Building E, 200 Lees Avenue, Ottawa, ON K1N 6N5 Canada; 2 School of Rehabilitation Therapy, Queen’s University, Kingston, Canada; 3 Department of Obstetrics and Gynaecology, Kingston General Hospital, Kingston, Canada; 4 Department of Obstetrics and Gynaecology, Foothills General Hospital, Calgary, Canada; 5 Department of Emergency Medicine, Kingston General Hospital, Kingston, Canada; 6 Department of Population Health Sciences, Queen’s University, Kingston, Canada; 7 Department of Obstetrics and Gynaecology, The Ottawa Hospital, Ottawa, Canada; 8 Department of Obstetrics and Gynaecology, Hôpital Montfort, Ottawa, Canada; 9 Department of Radiology, Kingston General Hospital, Kingston, Canada

**Keywords:** Pelvic floor muscles, Pelvic floor muscle training, Predictive model, Stress urinary incontinence, Transperineal ultrasound, Physiotherapy

## Abstract

**Introduction and hypothesis:**

The aim of this study was to prospectively identify aspects of baseline demographic, clinical, and pelvic morphology of women with stress urinary incontinence (SUI) that are predictive of cure with physiotherapist-supervised pelvic floor muscle training (PFMT).

**Methods:**

Women ≥18 years old with SUI were recruited from urogynecology and pelvic health physiotherapy clinics. Participants completed a 3-day bladder diary, the International Consultation on Incontinence Questionnaire Urinary Incontinence Short Form (ICIQ-UI-SF), a standardized pad test, manual assessment of pelvic floor muscle (PFM) strength and tone, and transperineal ultrasound (TPUS) assessment of their urogenital structures at rest while in a supine position and standing, and during contraction, straining, and coughing. Participants attended six physiotherapy sessions over 12 weeks and performed a home PFMT program. The assessment was repeated after the intervention; cure was defined as a dry (≤2 g) pad test.

**Results:**

Seventy-seven women aged 50 (±10) years completed the protocol; 38 (49%) were deemed cured. Based on univariate testing, four predictors were entered into a binary logistic regression model: ICIQ-UI-SF, PFM tone, bladder neck (BN) height in a quiet standing position, and BN height during a cough in a standing position. The model was significant (*p* < 0.001), accurately classifying outcome in 74% of participants. The model, validated through bootstrapping, performed moderately, with the area under the receiver operating characteristic curve = 0.80 (95% CI: 0.69–0.90; *p* = 0.00), and with 70% sensitivity and 75% specificity.

**Conclusions:**

Women with better bladder support in a standing position and less severe symptoms were most likely to be cured with PFMT.

**Clinical trial registration:**

#NCT01602107.

**Supplementary Information:**

The online version contains supplementary material available at 10.1007/s00192-020-04583-z.

## Introduction

Urinary incontinence (UI) affects up to 50% of women, the majority of whom have stress urinary incontinence (SUI) [1]: the involuntary leakage of urine during effort or exertion (e.g., coughing, sneezing, etc.) [2]. UI costs the Canadian healthcare system approximately $3.84 billion CAD [3] annually, while also taking a heavy toll on women both socially (embarrassment/shame) [4] and economically (management products, extra laundry, conservative interventions) [3].

Current interventions for SUI include surgery, pessaries, urethral bulking agents, and physiotherapist-supervised pelvic floor muscle (PFM) training (PFMT)—for which there is strong evidence and no associated adverse effects [5]. As such, PFMT is recommended as a first-line treatment for SUI [6]; however, only half of women with SUI are cured with PFMT [5]. Physiotherapy can also be difficult to access, have long wait times, and is not covered by most health insurance plans, often requiring supplemental health insurance or large out-of-pocket expenses [3]. Identifying factors that predict the success of PFMT interventions for women with SUI may help to streamline treatment, reducing costs to individuals, private insurers, and healthcare systems, while improving the patient experience.

Previous predictive models for PFMT outcomes among women with SUI have been generated using combinations of demographic and clinical assessments outcomes [7–10] or PFM function and morphology [11, 12]. However, findings have been inconsistent, and most studies have been limited by the use of retrospective data [7, 8, 10, 11], including more predictors than is recommended to avoid over-specification [8, 10, 11], and/or a lack of validation using statistical techniques or testing the model on a new sample of women [7, 9–11]. Further, some models have used clinically inaccessible tools such as MRI and dynamometry [12, 13].

Using a prospective study design and clinically accessible measures, we aimed to create a robust and practical predictive model of women’s success with PFMT, evaluated objectively through observation of a dry pad test after the intervention period.

## Materials and methods

This was a prospective interventional cohort study. Institutional approval from the appropriate ethics boards was obtained prior to initiating recruitment. Recruitment and data collection occurred in Kingston, Ottawa, and Calgary along with a concurrent registered randomized control trial (RCT) investigating the effects of pre- and post-operative physiotherapy on surgical outcomes for women with SUI [NCT 01602107] as well as participants recruited outside the RCT.

### Participants

Women with SUI who were ≥ 18 years old and who were surgical candidates for mid-urethral sling insertion were recruited into the concurrent RCT from the surgical waitlists at four participating urogynecology clinics, and only those randomized to receive a physiotherapy intervention were included in this study. Additionally, women with SUI were recruited for this study from the waitlists of two local physiotherapy clinics. In both cases, women were included if they had predominant symptoms of SUI with or without urgency incontinence or nocturia. Women were excluded if they were pregnant or had been pregnant in the previous 12 months, had fecal incontinence, took medications known to increase or relieve incontinence, had known neurological impairments, connective tissue disorders, or known or suspected detrusor overactivity, had undergone previous pelvic surgery to treat SUI, or had known pelvic organ prolapse (POP). Telephone screening confirmed eligibility and eligible women were given verbal instructions on completing a series of questionnaires, which were subsequently mailed to them: the International Consultation on Incontinence Questionnaire Urinary Incontinence Short Form (ICIQ-UI-SF), the International Consultation on Incontinence Questionnaire Female Lower Urinary Tract Symptoms (ICIQ-FLUTS), the Medical Outcomes Survey SF-36, and a 3-day bladder diary, although the results of the last three questionnaires were not used for the purposes of this study.

### Baseline assessment

Prior to the baseline assessment, women provided demographic information, and a medical history via telephone interview. Women were asked to void their bladder 1 h before the scheduled assessment and then to drink 500 ml of water at that time. At the assessment visit, the study physiotherapist reviewed the 3-day bladder diary to determine continued eligibility—women had to report at least one leakage episode attributed to a stress event over the 3 days, and no more than one UI event associated with urgency. Bladder volume was estimated using trans-abdominal ultrasound: once it was between 300 and 400 ml, participants completed a standardized 30-min pad test [13]. Women were excluded at this point if their pad test was dry (< 2 g). Women who remained eligible voided their bladder before the physiotherapist performed a physical examination, including sensory and reflex testing, evaluation of PFM strength (Modified Oxford Scale [MOS] score 0 to 5) [14] and PFM tone (graded between +3 and −3) [15], in that order. The sensory testing involved evaluating accurate reports of light touch to the S2–5 dermatomes, and reflex testing included confirming the presence of the anal wink reflex to rule out lower motor neuron lesion [16], which would have resulted in exclusion. The MOS was performed for the right and left sides of the levator ani separately, palpating with the first and second digits inserted vaginally and offering resistance in the 3 and 9 o’clock positions, separately as well as in the mid-sagittal plane, in the 6 o’clock position. Three maximal contraction efforts were performed following instruction to contract the PFMs as strongly as possible, squeezing and lifting against the resistance of the assessors fingers, and holding until instructed to relax, and was graded through palpation at each site; the median score of three trials performed at all sites was retained for data analysis. Tone was evaluated by rating resistance to passive elongation of the levator ani muscles through intravaginal palpation at the 3 and 9 o’clock positions as well as in the 6 o’clock position, and again the overall median value was retained. Participants then underwent a thorough two-dimensional (2D) and three-dimensional (3D) ultrasound assessment of their urogenital morphology (GE Voluson-*i,* GE Healthcare, Toronto, Canada).

For the ultrasound analysis, morphological features that have demonstrated evidence of an association with the pathophysiology of SUI were evaluated, including pelvic organ support [17], urethral mobility [18], urethral morphology [19], and PFM function [20]. First, with the woman in a relaxed lithotomy position, a mechanical sector 3D endoprobe (7.5 MHz) was placed at the external meatus of the urethra to visualize the entire volume of the urethra in the mid-sagittal plane following the protocol described by McLean et al. [21]. Three volumetric images were acquired, separated by 60 s between trials. Next, a 3D curvilinear probe (6.5–10 MHz) was applied transperineally to visualize the levator hiatus at rest and during maximum voluntary contraction (MVC) [22], and then to obtain 2D B-mode video clips of the pelvic structures (bladder, urethra, anorectal angle, and pubic symphysis) in the mid-sagittal plane at rest, during MVC, during maximal Valsalva (bearing down) maneuver (MVM), and during coughing. For the rest task, women were instructed to breathe in, then breathe out, then to remain quiet and relaxed, focusing on neither contracting nor relaxing their pelvic floor or any other muscles. For the MVC task, women were instructed to breathe in, then breathe out, then to lift their perineum and to squeeze around their anus and vagina as strongly as possible, as if to stop the flow of urine or the passage of gas and to hold the contraction until instructed to release it. For the MVM task, women were instructed to relax their PFMs, take a deep breath in, close their glottis, and then to bear down as forcefully as possible, as if forcing a bowel movement or delivering a baby. They were informed before each trial that the passage of urine or gas during the task was common, and would indicate that they were doing the task properly so that they would not hold back. Last, for the coughing task, women were instructed to perform a single-barrel cough as forcefully as possible. Three trials of each task were captured with a minimum of 60 s of rest between trials. The 2D imaging of the pelvic structures at rest, during MVM, and during coughing was repeated in a standing position, as the gravity-dependent position is more representative of the functional position in which urine leakage tends to occur.

### Physiotherapy intervention

The physiotherapy intervention was delivered by one of six physiotherapists, each with post-graduate training and > 5 years of experience in pelvic floor assessment and SUI management. The physiotherapy intervention consisted of six face-to-face sessions over a 12-week period: one session per week for the first 3 weeks, one session every 2 weeks for the next 2 weeks, and one final session 4 weeks later. The first session lasted approximately 45 min and subsequent sessions lasted 30 to 45 min. During these sessions, women were taught to perform proper PFM contractions, using electromyography biofeedback through an intravaginal probe at the first two sessions to promote motor learning, as well as manual therapy techniques when deemed necessary and appropriate to the woman’s particular situation. Women were also instructed to perform a strong PFM contraction before events that increase intra-abdominal pressure (the “knack”), and were prescribed a home exercise program through which they were instructed to perform three sets of ten maximum effort voluntary PFM contractions per day, with complete relaxation after each contraction. The exercises were reviewed and progressed at each session, including position changes (e.g., supine to standing, the speed and force of contraction, etc.). The full details of the intervention and exercise program are provided in Appendix 1.

### Follow-up assessment

Within 2 weeks of their last physiotherapy session, participants completed the 3-day bladder diary and the ICIQ-UI-SF and returned to the laboratory for a follow-up pad test, clinical assessment of PFM strength and tone, and ultrasound assessment of pelvic morphology. Women participating in the RCT continued to surgery for the purposes of that study whereas the follow-up assessment was the end of participation for women recruited outside of the RCT. The follow-up data were collected by the same research physiotherapist who performed the baseline assessment, and this individual remained blinded to whether or not participants received the physiotherapy intervention.

### Outcome variables

A cure with PFMT was defined as a dry pad test (i.e., pad weight gain ≤2 g) at the follow-up assessment [11]. Two-dimensional ultrasound image and video analyses were performed offline using ImageJ (NIH, Bethesda, USA) software, and 3D volume analyses were performed offline using 4D View® software (v.9; GE Healthcare, Canada). For all imaging outcomes, the median value of the three trials acquired for each variable and task were retained in the analysis. Morphological features of pelvic organ support included measures of the levator hiatus area in the plane of minimal hiatal dimensions (rest, supine, standing) [22], levator plate length (LPL; rest, MVM, coughing) [23], and bladder neck height relative to the levator plate line (rest, MVM) [22, 24] measured in the midsagittal plane. Features associated with urethral morphology included urethral length and urethral wall cross-sectional area [21]. Bladder neck and urethral mobility were assessed during coughing and MVM [22–24]. PFM function was measured as the change in levator hiatus area on MVC, change in LPL during MVC, and the most cranial position of the bladder neck relative to the levator plate achieved on MVC. Levator avulsion was assessed as described in Dietz et al. [25], using 3D volumes acquired during MVC. To enhance reliability, two independent raters assessed each 3D volume acquired from each participant and trial, and rated avulsion as present or absent based on the median rating across the three trials. If the raters did not agree, then an independent reviewer (LM or VD) evaluated the 3D volumes and discussed their ratings with the assessors until consensus was reached. See Appendices 2 and 3 for a complete list of the morphological variables measured.

### Statistical analysis

Participants who did not return for their follow-up assessment were excluded from the analysis as the post-treatment outcomes were essential to grouping women as cured or not cured in the model. All statistical analyses were conducted using SPSS 25. All variables were first tested for normality using the Kolmogorov–Smirnov test. To narrow the number of outcome measures for consideration as potential predictors of cure in a binary logistic regression model, univariate *t* tests and Mann–Whitney *U* tests were performed for normal and non-normal data; Chi-squared analyses—Fisher’s exact test as needed—were performed for categorical data. An α = 0.10 was set as a limit for inclusion in the predictive model. To avoid overspecification of the model, we planned a priori to include only one potential predictor from each category of morphological feature shown to be associated with SUI pathophysiology (pelvic organ support, urethral mobility, urethral morphology, and PFM function; see Appendix 2 and 3). Through univariate testing we identified the outcome variable with the largest effect size and that did not violate the assumptions of linear regression (i.e., linearity of the logit, multicollinearity, and independence of error) from each category for inclusion in the model, and entered these along with demographic and clinical assessment outcomes. Missing values were handled through multiple imputations prior to generating the predictive model. The final model was tested for goodness-of-fit (Hosmer–Lemeshow test) and its Global Likelihood Ratio. To control for model optimism, bootstrap validation of each imputed dataset was performed based on 1,000 samples with replacement to provide a 95% confidence interval range. Odds ratios were used to describe the independent associations of each predicting variable to the outcome, and the diagnostic ability of the overall model was assessed using a pooled receiver operating characteristic (ROC) curve generated by taking the mean value for each predicting variable across the multiply imputed datasets.

## Results

**Fig. 1:**
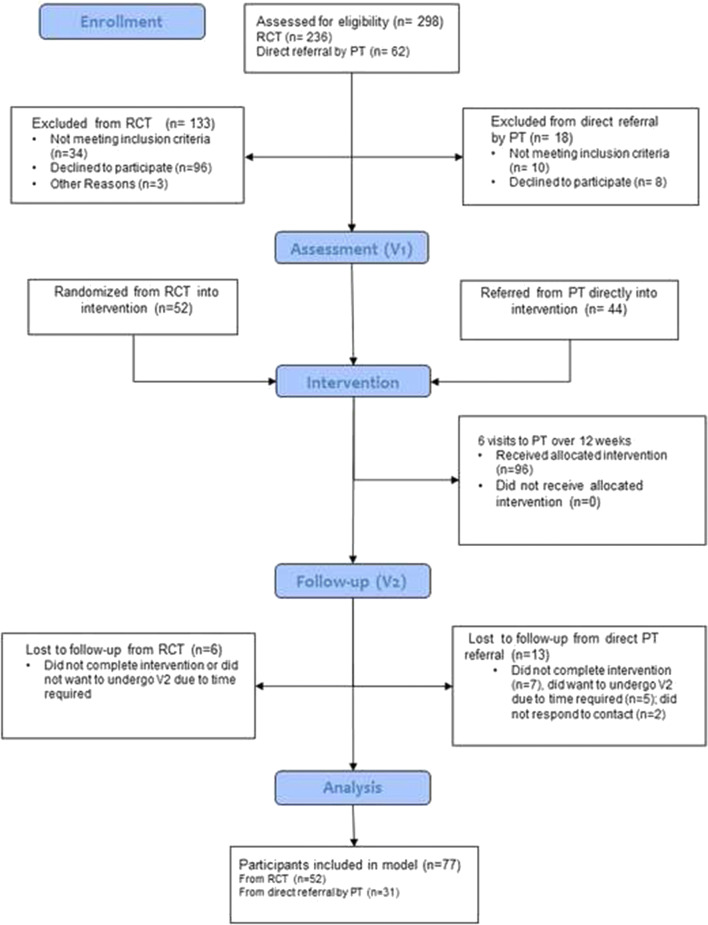
Consolidated Standards of Reporting Trials diagram. *PT* physiotherapy, *RCT* randomized controlled trial)

Recruitment and attrition are described in the CONSORT diagram presented in Fig. 1. Seventy-seven women completed the baseline and follow-up sessions and were included in the logistic regression model. There were no significant differences in baseline demographic or clinical outcomes between women who returned for their follow-up laboratory assessment and those who did not return. Tables 1 and 2 indicate sample demographic information, questionnaire results, and clinical assessment data recorded at baseline.

**Table 1:** Demographic, clinical assessment, and questionnaire data recorded at baseline (*n* = 77)

Demographics	Mean ± SD
Age (years)	50 ± 10
BMI (kg/m^2^)	28.27 ± 7.26
Parity (*n*)	3 ± 1
Weight of heaviest baby (kg)	4.12 ± 1.39
Baseline pad test (g)	20.81 ± 19.97
Baseline bladder diary (number of leakage episodes attributed to SUI over 3 days)	8 ± 7
Baseline ICIQ-UI-SF (scores from 0 to 21)	12 ± 4
Baseline PFM strength (scale from 0 to 5)	3 ± 1
Baseline tone (scale from −3 to +3)	1 ± 1

*BMI* body mass index, *ICIQ-UI-SF* International Consultation on Incontinence Questionnaire Urinary Incontinence Short Form, *PFM* pelvic floor muscle

**Table 2:** Categorical data recorded at baseline (*n=77*)

Demographics	Frequency n (%)
Smoking	
Yes	9 (12%)
No	68 (88%)
Menopausal Status	
Post	39 (51%)
Pre	38 (49%)
Hormone Replacement Therapy	
Yes	7 (18%)
No	32 (82%)
Hormonal Contraceptive use	
Yes	8 (21%)
No	30 (79%)
Hysterectomy	
Yes	10 (13%)
No	67 (87%)
Use of Forceps during Delivery*	
Yes	6 (9%)
No	62 (91%)
Use of Vacuum during Delivery*	
Yes	6 (9%)
No	62 (91%)
Number of C-Sections*	
0	58 (85%)
1	5 (7%)
2	4 (6%)
3	1 (2%)
Avulsion	
Yes	5 (6%)
No	72 (94%)

Note. *corrected to the percentage of parous women in the sample (*n* = 68), excluding the nulliparous women (*n* = 9)

Univariate analyses revealed 14 potential predictors (*p* ≤ 0.10) for inclusion in the logistic regression model (Appendix 2, 3, 4, 5). In particular, women who were cured with the PFMT intervention had evidence for better pelvic organ support than those who were not cured, demonstrating higher bladder neck height in a quiet standing position (*p* < 0.00), at rest in a supine position (*p* < .00), and during MVM in a supine position (*p* = 0.00); shorter LPL during coughing in a supine position (*p* = 0.03) and during MVM in a standing position (*p* = 0.04); smaller levator hiatus circumference at rest in a supine position (*p* = 0.04) and in a quiet standing position (*p* = 0.01), which was also noted in the levator hiatus area (*p* = 0.03). The women who were cured tended to have less urethral mobility, demonstrated by less bladder neck descent seen during a cough performed in a supine position (*p* = 0.03) and in a standing position (*p* = 0.03). Measures of urethral morphology, including a cross-sectional area of the urethral wall (*p* = 0.63) and urethral length (*p* = 0.97) showed no differences between cohorts. Two variables indicative of PFM function showed evidence for group differences when measured in a supine position, LPL at end MVC (*p* = 0.09) tended to be shorter, whereas bladder neck height at end MVC (*p* = 0.03) was significantly higher. Baseline clinical assessment variables that were different between the groups included the pad test (*p* < 0.00) and the ICIQ-UI-SF scores (*p* = 0.03), both of which indicated that the group deemed cured with the intervention had less severe incontinence than those who were not cured. Although PFM strength (*p* = 0.29) and the presence of levator avulsion (*p* = 0.80) showed no difference between groups, the women who were cured had higher PFM tone (*p* = 0.01) than those who were not cured with the intervention.

The potential predictors included in the model were: bladder neck height in a quiet standing position, bladder neck height during a cough in a standing position, PFM tone, and ICIQ-UI-SF score. There were eight missing data points, and as such, eight multiple imputations were generated.

The binary logistic regression model was significant (*p* < 0.001) with two significant predicting variables: SUI severity as measured by the ICIQ-UI-SF and bladder neck height measured in a quiet standing position. Two-way interactions between variables were tested, but none was significant. PFM tone (Block Chi-squared = 0.52, *df* = 1, *p* = 0.51) and minimum bladder neck height during a cough performed in a standing position (Block Chi-squared = 1.04, *df* = 1, *p* = 0.31) were removed because of their poor contributions to the model fit. The overall predictive model accurately classified 74% of women as either cured or not cured and the Nagelkerke generalized R^2^ was 0.35. The model fitted the data well (Hosmer–Lemeshow goodness-of-fit Chi-squared = 7.60, *df* = 8, *p* = 0.49) and the Global Likelihood Ratio was moderate (+LR = 2.53, −LR = 0.34). Participants in whom the bladder neck sat more cranially within the pelvis were more likely to have a dry pad test after the PFMT intervention than women with less bladder neck support (*p* = 0.001). Women with lower scores on the ICIQ-UI-SF were more likely to have a dry pad test after the PFMT intervention than women with more severe symptoms (*p* = 0.024). Table 3 displays the regression coefficients, odds ratios, and 95% confidence intervals for the predictive model.

**Table 3:** Odds ratios and confidence intervals for significant predictors of physiotherapy outcomes among women with SUI

Predictors	B	OR	Bootstrapped 95% CIs		*p*
			Lower	Upper	
Constant	−.936	.392	−3.52	1.97	.392
Baseline ICIQ-SF (scores from 0 – 21)	−.150	.860	−.31	−.02	**.024**
Bladder Neck Position in quiet standing	.197	1.218	.06	.49	**.001**

*Note. Odds ratio (OR); bolded values are significant (*p* ≤ .05); International consultation on incontinence questionnaire – short form (ICIQ-SF); 95% CIs (confidence intervals) are presented as the minimum lower value and the maximum upper value of the range of 95% CIs from each of the multiply imputed datasets

The diagnostic ability of the predictive model was assessed using an ROC curve. The area under the curve (AUC) for the model was 0.80 (95% CI: 0.69–0.90; *p* = 0.00), suggesting that the model effectively separates women who are deemed cured and not cured based on the ICIQ-UI-SF scores and the position of the bladder neck using Eq. 1 with 70% sensitivity and 75% specificity.

For predicted success with the intervention, the logistic regression equation was:

1 $$ 0.2049\times \left( Bladder\ Neck\ Position\kern0.5em in\  mm\right)-0.1381\times \left( ICIQ\_ SF\right)\ge 1.0812 $$

**Fig. 2:**
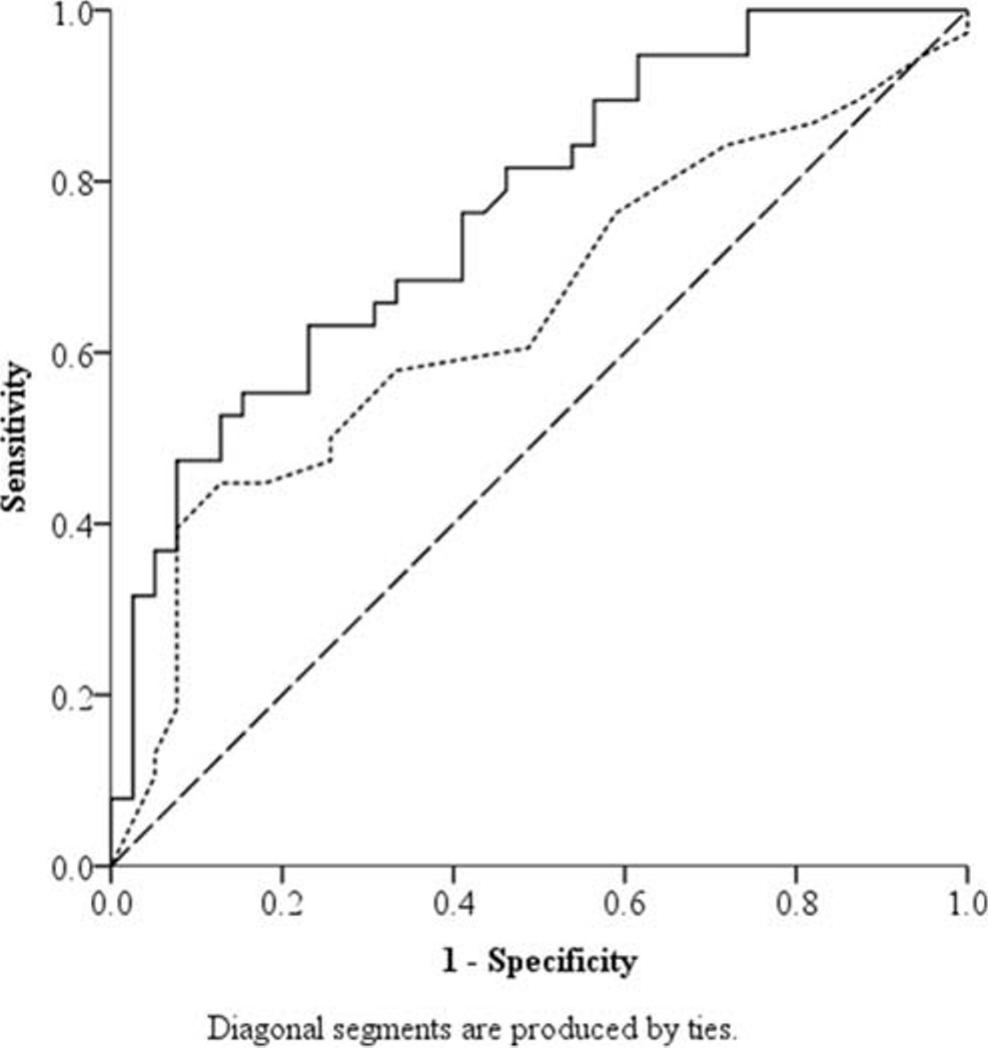
Receiving operator characteristic of separate predictors for the predictive model

For the individual predicting variables in the model, the AUC for baseline ICIQ-UI-SF was 0.65 (95% CI: 0.52–0.77; *p* = 0.03) and for the baseline bladder neck position at rest in a standing position was 0.77 (95% CI: 0.66–0.87; *p* < 0.01). Cut-off scores were estimated conservatively, balancing both sensitivity and specificity for each predictor. A cut-off of ≤10.5 (out of 21) on the baseline ICIQ-UI-SF score predicts PFMT outcome with 45% sensitivity and 85% specificity; a cut-off height of ≥14.3 mm for baseline bladder neck position in a standing position predicted PFMT outcome with 65% sensitivity and 75% specificity (Fig. 2) When the model was reversed to predict which women with SUI were less likely to be cured by a PFMT, the predicting variables remained the same, as did the estimated cut-off scores from the ROC curve with similar specificity and sensitivity.

## Discussion

The final predictive model for success with the PFMT intervention was significant, with two predictors: bladder neck height in a quiet standing position and self-reported SUI severity, such that women with a more cranially positioned bladder neck during quiet standing and those with lower SUI severity as measured by the ICIQ-UI-SF were more likely to be successful with PFMT. Based on the sample size, our model was not over-specified and is the first, to the authors’ knowledge, to combine morphological predictors with clinical and demographic variables. These data support the current recommendation that women with mild to moderate SUI try physiotherapy as a first-line intervention [6], but adds that women with better pelvic organ support, measured using ultrasound imaging performed in a standing position, are more likely to have better outcomes.

Several previous studies have generated predictive models of PFMT outcomes among women with SUI using various definitions of cure [7–12]. As there is currently no standard definition of cure available in the literature, we used the same definition employed by Dumoulin et al. [11]: a pad weight gain of ≤2 g on a standardized pad test post-treatment. Using this criterion, the cure rate found in this study was 49%, which is within the range of cure rates reported in previous studies 36–74% [7, 8, 10–12, 26]. Although other definitions of cure may yield different cure rates and results in predictive modeling, the use of a dry standardized pad test to operationalize the definition of cure is objective and easily replicable.

Similar to our findings, Cammu and colleagues [9] found that physiotherapy intervention was more likely to fail in women who reported more severe symptoms (> 2 leakages per day). These findings are consistent with observations that women with mild to moderate SUI severity are more often cured with physiotherapy in comparison with their more severely suffering counterparts [8]. Women with more severe SUI symptoms may have more defects or more extensive defects including tissue damage, nerve damage, and/or reduced vascularization of their urethra and/or pelvic floor, making a complete cure more difficult to attain through conservative approaches. PFMT interventions may provide some compensation for these defects [27], but may be insufficient when the defects are more severe.

Bladder neck height reflects the support of the bladder and trigone, and as demonstrated by the ROC curve, bladder neck height in a quiet standing position holds most of the predictive ability of this model. In this study, women whose bladder necks were positioned more cranially within the pelvis were more likely to be cured by the physiotherapy intervention, which is consistent with Dumoulin et al. [12], who found that an MRI-based measure of bladder neck support also significantly predicted the outcome of physiotherapy treatment for women with persistent (6 months) post-partum SUI. Unlike MRI, where two bony landmarks are available from which to make measurements, the limited viewing angle on ultrasound forces the measurement of bladder neck height to be relative to the levator plate, which is not a fixed landmark. Despite this limitation, the results obtained here suggest that this measure might be sufficiently sensitive to detect differences in connective tissue support of the bladder and trigone. Indeed, women with more severe strain of the connective tissues supporting the bladder and urethra may be less successful with an exercise intervention, as PFMT may compensate for these defects but cannot correct them.

Despite the finding that women with SUI who were cured with the intervention had higher PFM tone at baseline than women who were not cured, in the current study, PFM tone did not contribute significantly to the predictive model. Unlike our model, using a custom intravaginal dynamometer Dumoulin et al. [11] found that women with persistent post-partum SUI who had lower resistance of the PFMs to passive stretch (e.g., lower tone) at baseline were significantly more likely to benefit from a physiotherapy intervention. Although this difference in results may be due to subjectivity or a lack of sensitivity of digital palpation to assess PFM tone [15] in comparison with dynamometry [28], it is more likely that this difference is due to a difference between the study samples: the patients in our sample were much older and had SUI that had persisted for much longer. More information about passive PFM tissue properties is needed in order for us to understand their relevance to the pathophysiology of SUI, as well as to the success or failure of physiotherapy interventions for SUI in women.

Urethral hypermobility is a contributing factor in SUI, such that women with SUI demonstrate greater urethral motion and acceleration during coughing [18]. However, in this study the extent of bladder neck descent seen during a cough, which reflects the aspects of connective tissue length and strength, was not a significant predictor in the model. While women who were cured with the intervention demonstrated more constrained descent of the bladder neck during coughing than those who were not cured, this variable may be confounded by PFM strength, power, or motor control: a cranial lift and squeeze of the PFMs during coughing may prevent the bladder (and other pelvic organs) from descending below the levator plate [19], even in the presence of significant strain to the connective tissue support. The predictive value of this variable may have been limited because some women may naturally adopt the compensatory strategy of the “knack” whereas others may have learned this strategy through the intervention.

There was no difference between the groups on avulsion status; however, the sample only included 5 women with levator avulsion. The presence of a levator avulsion changes the morphometry and can impair the function of the PFMs, especially during the early postpartum period [29]. However, even with the impairments in PFM strength, speed of contraction, and endurance [29] avulsion does not appear to be directly linked to SUI [30], but is more relevant to POP [31]. This may explain why so few women with SUI in this study demonstrated evidence of levator avulsion on ultrasound. Although the results of this study suggest that the presence of levator avulsion might not be related to treatment outcome among women with SUI who receive physiotherapist-supervised PFMT, further research is needed to confirm this.

The measures included in our model to assess the likelihood of success with PFMT among women with SUI are easily replicable in clinical settings. Specifically, the ease of measuring bladder neck height in a quiet standing position makes it a useful, translatable clinical assessment to help streamline treatment selection. 2D imaging is readily available in many centers, involves less expensive instrumentation and less technical expertise than 3D imaging, and does not depend on the specific instructions to the patient or on their capacity to perform the task correctly, as is the case with MVC, cough, or MVM.

The results of this study are generalizable only to women who have SUI without significant prolapse (i.e., grade II or less on the POP-Q), who have no concurrent fecal incontinence, and who have not previously undergone physiotherapy or surgical intervention for their SUI. We did not include a measure of bladder neck dilation in this study, despite the fact that it has been shown to be strongly associated with the presence of SUI in women [32]. Although this may be considered a limitation, Dumoulin et al. [12] did test urethrovesical junction approximation during straining and found it not to significantly predict PFMT outcomes among women with persistent post-partum SUI. It is possible that this variable does have predictive value in older women, and this should be investigated in future work.

Another limitation to this study is that the follow-up assessment was done in the short term, 2 weeks after completing the 12-week intervention. This constraint was necessary as most participants in this trial were enrolled in a concurrent RCT through which they were scheduled for mid-urethral sling insertion following the intervention, and therefore long-term follow-up was not possible. Although results may differ if modeling factors predictive of long-term success with PFMT, success with PFMT has been found to be sustained in the long term in 41–85% of women, regardless of their adherence to the exercises after completing the original intervention [33].

The recruitment of participants from two different settings (i.e., from surgical wait lists at urogynecology clinics (inside the concurrent RCT) and from physiotherapy clinics (outside of the concurrent RCT) was necessary in order to generate an adequate sample size and also to ensure that the sample had a wide range of baseline characteristics. This approach was unlikely to introduce bias, as all participants were recruited prospectively using the same inclusion/exclusion criteria, were evaluated by the same, blinded, research physiotherapist at both time points, and underwent the same intervention with the same affiliated physiotherapists.

Further model validation on a new group of women with SUI should be completed before translating it to clinical practice. Future work should investigate other variables that may improve the performance and robustness of the model.

## Conclusions

The model of success with a PFMT intervention among women with SUI revealed two significant predictors: SUI severity, as measured by the ICIQ-UI-SF, and bladder neck height measured in a quiet standing position through 2D transperineal ultrasound. Women with lower symptom severity and a more cranial bladder neck position in quiet standing were more likely to demonstrate a dry pad test after the intervention. Indeed, bladder neck support in quiet standing accounted for most of the predictive ability of the final model. The results of this study support current clinical recommendations that women with mild to moderate symptoms may be better suited to physiotherapy interventions for SUI than are women with more severe symptoms. They add to this a recommendation that evaluation of the bladder neck position in standing may be useful for clinical decision making. The predictive model presented here is novel in that it was generated using prospectively acquired data, considered demographic, clinical, and morphological variables. With further revisions and validation of this model, the results of this study may help to guide treatment recommendations for women with SUI.

## Supplementary information

ESM 1 (DOCX 121 kb)
