# Supplementary material for: A model identifying characteristics predictive of successful pelvic floor muscle training outcomes among women with stress urinary incontinence
Source: Int Urogynecol J. 2020 Nov 25;32(3):719–28. doi: 10.1007/s00192-020-04583-z (PMC7902568; doi:10.1007/s00192-020-04583-z)
Supplement: Supplementary file 1 — (DOCX 121 kb) [file 192_2020_4583_MOESM1_ESM.docx]

Appendix 1

**PHYSIOTHERAPY (PT) TREATMENT PROTOCOL**

*Note: Participants are informed in written form and verbally of their right to have a person of their choice also present during PT sessions. Prior to treatment, before each treatment session, the therapist will discuss with the participant treatment components and the plan of treatment for the session and obtain verbal consent from the participant. The participant is also asked to hand in a weekly exercise log, if they have not done so, they are asked to complete one based on what they remember doing in the past week.*

**GUIDELINES:**

- The following protocol outlines the different PT treatment components, goals, descriptions, and the parameters, and the documents related to treatment that will be used.

**TREATMENT SPECIFICATIONS:**

- Number of treatment sessions: 6 sessions
- Treatment frequency: one session/weekX2 weeks, 1 session/2 weeks X 8 weeks
- Total treatment time: 10 -12weeks (assuming each participant will have two weeks where a scheduled visit is either not possible or cancelled)
- Expected duration of each session: First session 1 hour, subsequent sessions 30-45 minutes

**TREATMENT COMPONENTS:**

***Each session will involve three components:***

***1. Education***

***2. Review of previous exercise and reassessment of PFM strength***

***3. Progression of the exercise program.***

***1. Education***

Education is provided by the treating physiotherapist in order to inform, situate and involve the participant in their care. The education that will be provided is listed below in point form.

1) Functional anatomy**:** Explain the functioning and anatomy of pelvic floor muscles (PFMs) using educational tools (drawings, 3D pelvis anatomical model), including explaining their role in vaginal, urinary, and anal functioning (canister; diaphragm, PFMs - breathing, lumbopelvic stability, abdominal muscle co-ordination etc.). Continence mechanism; impact of intra-abdominal pressure

2) Different types of UI: urge, stress, mixed.

3) Types of prolapse

4) Prevalence of SUI (approx. 40% of women in general population) and prolapse (even more common)

5) SUI = Not normal aging process

6) The importance of improving strength and motor control of PFMs.

7) Two support systems: fascia and muscles

8) Goals of physiotherapy: The main goal is to decrease occurrence of urine leakage by increasing strength, awareness and control of their PFMs with active PFM exercises (i.e. Kegel exercises), biomechanics, educate and provide information, help the patient gain control over the condition.

9) Physiotherapy treatment components**:** education, bladder diary, active PFM exercises (i.e. Kegel exercises; speed, endurance, strength and graded contractions), biofeedback PRN, manual stimulation/feedback PRN, proprioception and myofascial techniques if appropriate, at-home program including log diary to control and increase compliance and recommendations on bladder training, posture assessment, lombo-pelvic assessment, breathing assessment, etc., combined PFMEs with ADLs, functional act..

10) Expected outcomes**:** so far studies show a success rate ranging from 40-80% amongst women who undergo physiotherapy treatment.

11) Provide information on possible causes of SUI – tied to anatomy, and prolapse (surgery, birth, bad voiding habits, overweight, straining, etc.).

12) Bowel habits**:** bowel routine, avoiding straining, nutritional advice

13) Bladder training:

- - - Avoid high impact activities. – suggest alternatives, waiting to see if problems improves... progressive return
    - Solutions to avoid straining (evacuation (handout) and constipation protocol, resources if applicable)
    - Lifestyle choice: amount of water drank daily, before bed, etc.
    - Weight reduction – if applicable – mention correlation weight and Pelvic organ prolapse and SUI and offer resources
    - Scheduled voiding (retraining frequency) – provide pamphlet, postponing 10-15min. – if applicable
    - Types of liquids and bladder irritants
    - Avoid exercises before and during voiding

14) Discuss adherence/compliance, and importance of exercises and motivation, etc. PT as a coach. Stress importance of honesty about exercise frequency and intensity- mention that this is an important study outcome! Stress importance of attending treatment even if participant has not done the exericises.

15) Postures during work and activities of daily living – stress importance of posture and proper biomechanics to avoid high intra-abdominal pressure

Once the treatment is initiated, the physiotherapist asks for **feedback** from the participants on the home exercises and reviews their log diary to look at **progression** by observing how many times exercises were performed, if they were done to completion. Tied with progression based on manual assessment during treatment- objective assessment.

The therapist and the participant discuss and formulate goals for the each treatment session based on the progression of the participants so far.

At all sessions the therapist will enquire about and address patient’s questions and concerns.

Total education time at 1^st^ session: 20 min.

Total education time at subsequent sessions: 5-10 min.

*Note: educating, answering questions posed and addressing concerns is continuously done through-out treatment sessions even during other components listed below.*

***2. Re-assessment and review of previously prescribed exercises***

1) Home exercise program and weekly log reviewed (Appendix 6). If participant forgets to bring the log, review their weekly exercise activities at that visit.

2) The therapist watches the patient perform her PFM exercises– using palpation and observation she ensures that the patient is performing the exercises properly. (See below; for exercise prescription)

3) Assessment of PFM strength, tone and endurance [88]

***3. Pelvic floor muscle (PFM) exercises***

*Note: Research into the prescription of PFM exercises is limited at this point despite the fact that several RCTs have found that PFM exercises are beneficial to relieve symptoms of SUI [7]). Despite the fact that in most studies of PFM exercise, the prescription exceeds the parameters that are recommended for other skeletal muscles, these parameters have been found to be beneficial for women with SUI. In particular, women are encouraged to perform three sets of maximal effort contractions three times per day and to perform these exercises daily. In other skeletal muscles, such a prescription would result in overtraining and strength losses. The fact that this prescription works for the PFMs may have a lot to do with the fact that women do not seem to be able to contract their PFMs maximally using voluntary contractions [109], and that most women will not actually perform the exercises daily even though that is what is prescribed.*

**Guidelines:** Participants are instructed to practice three of the following PFM exercises to improve the strength, endurance and motor control of her PFMs in different positions and circumstances every day. All participants begin all exercises in the supine or crook-lying position and each exercise is progressed from supine to reclined sitting to sitting and then to standing. At the first treatment session, all participants begin with exercise 0 to ensure that they learn a proper PFM contraction (approximately 50% of women cannot perform a proper PFM contraction with verbal instruction and will require some form of biofeedback) and then they are prescribed exercises 1, 2 and 3 in the first week. They will be instructed to perform 2 sets of 10 repetitions of each exercise daily, and to progress to a reclined sitting and a sitting position over the course of the week if they are finding that the exercises are easy to perform and are going well. At their next visit, the PT will assess their performance of the exercises in supine and then in sitting to ensure that they are being performed properly and will then progress the exercises accordingly. At the first session, the participant will also learn “the Knack” (Exercise 6; Miller et al.) and will be instructed to contract their PFMs throughout their day whenever they are about to change positions, cough, sneeze or laugh. They will be encouraged to continue this behaviour indefinitely.

Throughout the 12 weeks of treatment, participants will continue to perform exercise 1, the basic contract-relax, for two sets per day. This exercise may be progressed to being performed in different positions or during different tasks (e.g. Exercises 8, 9, 10). All participants will progress to exercise 4 once they can perform two sets of ten repetitions of exercise 1, since a quick contraction is thought to be very important functionally. Exercise 4 can also be progressed to functional positions (Exercises 8, 9, 10) once the participant has mastered the ability to perform 2 sets of 10 repetitions in supine. All participants will be progressed to exercise 7 to develop some endurance in their PFM contraction, and, again, once they can perform exercise 7 with a 45 second hold, they may progress to performing this exercise during functional tasks. It is expected that by the end of the 12 week physiotherapy program, all participants will be performing exercises 1, 4, and 7 daily in the sitting and/or standing position, and they will be advised to continue to practice these exercises until their surgery, to begin them again after their surgery and to continue to practice them until 5 weeks after their surgery. At that point they will be advised that they can perform the exercises 2 or 3 days per week to maintain their status.

There are three guiding principles to exercise prescription and progression. The first guiding principle is that the patient has to be reasonably challenged based on their progress. E.g. If patient can perform 4 good rapid contractions over 10 seconds.

**Exercise of the week**: Perform 5 contractions over 10 seconds. The second guiding principle is that a participant will not perform any exercises if they are prescribed too many, so a maximum of three PFM exercises along with some form of cardiovascular or postural training will be prescribed each week. The PFM exercises should take no longer than 10 minutes to perform. The endurance and postural correction exercises will be prescribed above and beyond that, but the participant will be instructed to focus first and foremost on the PFM exercises. A third guiding principle is that a participant will not perform exercises if they perceive them to be too easy or not to be necessary. As such, the participant will discuss their perception of the exercises with the therapist and exercises will be prescribed to maximize the likelihood that they will be performed.

**Progression:** Progress can be considered an increase in number of repetitions or sets, a change in position, or the addition of a functional activity to be performed concurrently with the PFM exercise. Progression of the exercises is prescribed on an individual basis and is based on the participant’s strength as assessed at the start of each treatment session as well as based on their feedback and demonstrated performance of the exercises prescribed in the previous week.

**Cues:** To perform a contraction of their PFMs, participants are instructed to “tighten” “pull” “lift” or “squeeze” as if they were trying to hold urine or avoid the passage of gas. To perform a relaxation of their PFMs, they are instructed to “let go” completely of the contraction, “relax” and “not do anything at all”.

| **Exercise** | **Goals** | **Instructions/Details** | **Parameters** |
| --- | --- | --- | --- |
| **0.Initiation PFME** | - To feel the PFM lifting motion in supine and then in standing | **-** Instructed to stand over a chair’s armrest and to lift the PFMs and feel the change in pressure in standing | - 1 set = 10 reps |
| **1. “The basic Contract-Relax”**  - this exercise will be prescribed to all participants | - Identification of PFMs  - Identification of the contrast between contraction & relaxation of PFMs | - A maximal voluntary contraction followed by a complete relaxation of their PFMs which needs to be at least twice as long as the contraction phase to allow full relaxation  - Variance: focus can be put on contraction or relaxation or both | - Contraction held 2 seconds, relaxation for a minimum of 2 seconds  - 1 set = 10 repetitions of contraction and full relaxation  - 3 sets/day |
| **2. The “Wave”**  -this exercise will be prescribed if the participant is not able to achieve a strong PFM contraction and has poor awareness of her PFMs.. | ↑ awareness of PFMs  - Isolation of different regions of the pelvic floor  - Isolation of PFMs vs. other muscles such as abdominals, gluteals, hip adductors | - A contraction focusing on one region of the PFMs at a time, beginning by focusing on contracting around the anus, then the vagina, and then the urethra. Then, do the reverse, by beginning at the urethra, vagina, and then anus.  - Thus, the movement is like a wave going back and forth.  (admittedly women cannot actually contract separate regions individually but the process helps them to become aware of the sensation of the PFMs contracting) | - Contractions at each section are held for 2 seconds, followed by a relaxation of 4 seconds before moving on to another section.  - The start direction is unimportant, but each direction of movement from front to back, or back to front is repeated 5 times.  - 1 set = a total of 10 contractions at each section (anus, vagina, urethra)  - 2 sets/day |
| **3. The “Elevator”**  **-** this exercise will be prescribed if the participant can initiate the contraction but has difficulty generating a strong contraction, or does not relax the PFMs well after contraction | - ↑ control of contraction and relaxation PFMs | - A gradual contraction and a gradual relaxation of the PFMs.  - Women are asked to imagine that their PFMs are like an elevator going up from the ground-floor (GF) to the 3^rd^, where the GF is complete relaxation and 3^rd^ floor is maximal contraction.  - They are asked to contract/go up one floor at a time to the 3^rd^, and relax/go down one floor at a time to the GF. | - Each contraction step up or relaxation step down is held for one slow count, about 2 seconds, including the 3^rd^ floor.  - Each ground-floor relaxation is held 5 seconds  - 1 set = going up and down the 3 floors 5 times  - 2 sets/day  ** can be used to retrain relaxation in certain cases |
| **4. “Quickies”**  **-**this exercise will be prescribed to all participants after they have mastered exercise 1. | - ↑ control of full relaxation after a quick contraction  - allow efficient response to prevent leakage effectively  - increase motor control and speed  - leading to THE KNACK exercise | - A series of quick contractions with a prompt and complete relaxation between each contraction.  - The emphasis is on the relaxation, and trying to attain the best relaxation as promptly as possible.  - Before contracting, the participant must fully relax, thus the contractions will be much quicker than the duration of relaxations. | - Each contraction is held for about 1 second, & relaxations depend on how quickly the participant can relax fully.  - 1 repetition = 5 quick contractions and relaxations  - 1 set = 5 repetitions, with 10 seconds of rest between each set  - 2 sets/day |
| **5. “The squeeze” (ie., maximum voluntary contraction + extra squeeze)**  **-**this exercise will be prescribed to participants who do not reach maximal contraction OR for whom “the knack” is not effective in reducing urine loss. | - ↑ strength and build muscle mass  - work in inner range of PFMs | - A series of strong maximal contractions held for 5s followed by a 5s extra contraction – called peaks or ROOF (if using the elevator analogy).  - The emphasis is on contracting the urethra, vaginal and anal opening simultaneously.  - Special attention given to avoiding contraction of the buttocks and thighs.  - Rest period must be respected between sets to avoid for muscular recovery. | - Each contraction is held for about 5 second and if possible, up to 5 peaks are added to the contraction.  - 1 series = 5 contractions or 5 seconds, with 10 seconds rest in between contractions  - 1 set = 3 series, with 2 minute of rest between each series  - 5 sets/day |
| **6. “The knack”**  **-**this exercise will be prescribed to all women as a behaviour modification but may also be prescribed as a specific strengthening exercise if the women is not remembering to perform a pre-contraction before activities that increase intra-abdominal pressure | - ↑ motor control and speed in a functional setting.  - To “Reset” the required automatic contraction that occurs before an increase in intra-abdominal pressure to prevent leakage. | - The patient is instructed to contract their PFMs before lifting, pushing, sneezing, laughing, etc. | - Attempted in supine than in standing.  - Performed at every occurrence of increase IAP pressure. Included in ADLs.  - Instructed to attempt it by pretending to cough.  - 1 set = 5 times  - 2 sets/day |
| **7. The “marathon”**  -this exercise will be prescribed to all women after they have mastered exercise 1. | - ↑ endurance | - The patient is instructed to hold the contraction as long as possible (starting at 10 seconds, and building up to 45 seconds by the end of the treatment). | - Each contraction is held for as long as possible (at least 10 seconds to start) until the patient can do 45 seconds.  - 1 set = 3 contractions  - 3 sets/day |
| **8. PFM during sit-to-stand**  **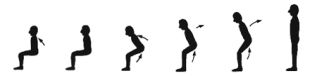** | - PFM contraction during a task (ie, getting up from toilet to avoid post-voiding dribbling) | - As demonstrated.  -Patient instructed to try to contract PFM and keep contraction while standing up.  - Can be performed at the toilet after voiding. | -Progression of basic exercises (1-7) as appropriate. |
| **9. PFM during squat**  **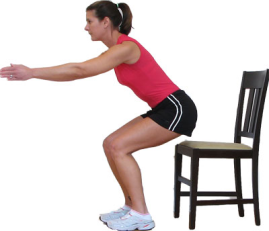** | -To help the patient develop PFM control in an abducted position. | Variance: less deep, hands on waist, hands on chair for support, etc. | - Progression of basic exercises (1-7) as appropriate. |
| **10.. PFM + abdominal and lower extremity exercises** | -Exercises usually performed in half sitting or supine that combines all muscle groups for core strengthening. | -Instructed to contract PFMs and Transversus Abdominus while supine with knees bent and to let one leg open out while holding the contraction.  -Progressed to active straight leg raise while holding, | -Progrssion of exercises 1-7 as appropriate. |
| **11. General cardiovascular conditioning walking, swimming, etc.** | Exercise prescribed for all participants based on their initial activity level |  | - instructed to perform a fast paced walk beginning at 20 minutes per day and to progress to longer duration or faster speed depending on the participant’s progress. |

**Additional therapeutic techniques employed during treatment:**

**1. *Manual therapy techniques – performed on 1^st^ treatment and then as appropriate***

**Goals:** Increase proprioception/awareness of the PFMs, mobilise PFMs and soft tissue (fascia and skin) by increasing circulation, stimulate and facilitate PFM contractions, strengthen the PFMs, help with relaxation, desensitize and release tension.

**Guidelines:** All manual techniques are performed by the physiotherapist with 1 or 2 gloved and lubricated (for intra-vaginal techniques) fingers. The use of 1 or 2 fingers depends on the participant’s level of comfort. Not all manual techniques need to be used with all participants, techniques are chosen based on participant findings upon assessment.

1. ***Manual resistance***

**Goal**: Offer resistance to difference levator ani muscles to help address specific weakness in the pelvic floor in order to build strength and increase proprioception.

**Description**: Moderate to high pressure is applied using one or two fingers (more pressure is applied with two fingers), inside the vagina on PFMs anywhere points of weakness if found. Resistance is offered for 5 seconds while the patient contracts.

**Progression**: Amount of pressure applied can be gradually increased according to the participant’s level of comfort and strength.

1. ***Proprioceptive stimulation / “Tapping”***

**Goal**: To increase PFM awareness/proprioception in individuals who are having difficulty learning a proper PFM contraction

**Description**: Gentle tapping is done inside the vagina on PFMs, after which the participant can then be asked to perform a PFM where she feels the tapping. The tapping can also be done externally, for example over the central perineal tendon.

1. ***Proprioceptive neuromuscular stimulation***

**Goal**: To increase PFM awareness/proprioception and facilitate PFM contractions in individuals who cannot generate adequate force or cannot initiate the contraction

**Description:** A quick, light stretch is performed on PFMs to elicit involuntary reflex contractions which may allow the participant to better perceive PFMs contractions.

**2. *Biofeedback and exercises – performed as needed. This approach is used to begin exercises if the initial strength grade is 0 or 1 out of 5.***

Biofeedback is performed for all patients at the initial assessment - using the ultrasound system. For all weeks where the participant still scores a grade of 0 or 1 out of 5, an electromyography (EMG) biofeedback system is used to allow the participant and the physiotherapist to visualize the quality and duration of PFM contraction and relaxation.

**Goal:** Assist in pelvic floor muscle retraining, increase control of the PFMs.

**Description:**

- - - - The US probe is lubricated with a gel-like, water-based, conductive lubricant, covered with a condom and with more gel. It is applied over the lower abdomen such that the bladder base is visible. Patients are instructed to elevate the bladder base, by watching the imaging screen.
      - Instruction and encouragement are provided as necessary by the physiotherapist throughout the entire biofeedback session. This may include concurrent digital palpation and pressure feedback if necessary.

Appendix 2

Table 1. Univariate parametric analyses of group differences in pelvic morphology between women with stress urinary incontinence who were cured vs not cured with the physiotherapy intervention.

| Outcome Measure | Position & Task | Mean ± SD | | | | Missing (n) | Cohen’s *d* | *p* |
| --- | --- | --- | --- | --- | --- | --- | --- | --- |
|  |  | n | Cured | n | Not Cured |  |  |  |
| Pelvic Organ Support Measures | | | | | | | | |
| Height of BN (mm) | Supine Rest | 38 | 24.29 ± 3.98 | 37 | 21.16 ± 4.90 | 2 | .12 | **.00** |
|  | Standing Rest | 37 | 16.86 ± 5.42 | 35 | 11.09 ± 5.50 | 5 | .18 | **.00** |
| LH Area (mm^2^) | Supine Rest | 37 | 14.49 ± 3.07 | 39 | 15.57 ± 3.23 | 1 | .06 | .14 |
|  | Standing Rest | 38 | 17.57 ± 3.26 | 38 | 19.31 ± 3.36 | 1 | .03 | **.03** |
| LH Circumference (mm) | Supine Rest | 37 | 14.82 ± 1.62 | 39 | 15.60 ± 1.58 | 1 | .08 | **.04** |
|  | Standing Rest | 38 | 16.15 ± 1.66 | 38 | 17.14 ± 1.35 | 1 | .11 | **.01** |
| LPL (mm) | Supine Rest | 38 | 51.11 ± 7.68 | 37 | 53.93 ± 8.57 | 2 | .06 | .14 |
|  | Standing Rest | 38 | 57.61 ± 8.46 | 36 | 59.55 ± 7.39 | 3 | .04 | .30 |
|  | Supine Cough | 38 | 49.84 ± 9.29 | 38 | 54.79 ± 9.70 | 1 | .09 | **.03** |
|  | Standing Cough | 37 | 54.44 ± 9.90 | 37 | 57.44 ± 9.01 | 3 | .05 | .18 |
|  | Supine MVM | 38 | 52.63 ± 10.43 | 36 | 56.14 ± 9.58 | 3 | .06 | .14 |
|  | Standing MVM | 37 | 59.58 ± 10.43 | 36 | 64.46 ± 9.85 | 4 | .08 | **.04** |
| Urethral Mobility | | | | | | | | |
| BN Excursion (mm) | Supine Cough | 38 | 13.19 ± 5.41 | 36 | 13.78 ± 4.75 | 3 | .02 | .62 |
|  | Standing Cough | 37 | 11.65 ± 3.59 | 37 | 11.42 ± 4.95 | 3 | .01 | .31 |
|  | Supine MVM | 38 | 14.86 ± 5.57 | 36 | 15.00 ± 6.87 | 3 | .00 | .92 |
|  | Standing MVM | 37 | 13.55 ± 4.81 | 36 | 12.28 ± 5.84 | 4 | .04 | .31 |
| Urethral Morphology | | | | | | | | |
| Urethral Length (mm) | Supine Rest | 37 | 3.13 ± .56 | 36 | 3.14 ± .80 | 4 | .00 | .97 |
| Urethral CSA (mm^2^) | Supine Rest | 37 | 1.31 ± .38 | 36 | 1.27 ± .30 | 4 | .02 | .63 |
| PFM Function | | | | | | | | |
| LPL (mm) | Supine MVC | 38 | 43.90 ± 6.50 | 38 | 46.51 ± 6.67 | 1 | .07 | **.09** |
| LH Area (mm^2^) | Supine MVC | 38 | 13.36 ± 3.23 | 39 | 14.27 ± 2.92 | 0 | .05 | .20 |
|  | Standing MVC | 38 | 16.65 ± 3.52 | 37 | 17.12 ± 3.34 | 2 | .02 | .98 |
| LH Circumference (mm) | Supine MVC | 38 | 14.01 ± 1.84 | 39 | 14.67 ± 1.75 | 0 | .06 | .11 |
|  | Standing MVC | 38 | 15.63 ± 1.81 | 37 | 15.85 ± 1.72 | 2 | .02 | .95 |

*Note. Bladder neck (BN); Levator hiatus (LH); Levator plate length (LPL); Cross-sectional area (CSA); Maximal voluntary contraction (MVC); Maximal Valsalva maneuver (MVM); Cure is defined as 2g≥ on a pad test.

Appendix 3

Table 2. Non-parametric univariate analyses of group differences in morphological variables between women with SUI who were cured vs not cured with the physiotherapy intervention.

| Outcome Measure | Position & Task | Median (Interquartile Range) | | | | | Missing (n) | *r* | | *p* |
| --- | --- | --- | --- | --- | --- | --- | --- | --- | --- | --- |
|  |  | n | | Cured | n | Not Cured |  |  |  |  |
| Pelvic Organ Support | | | | | | | | | | |
| Height of BN (mm) | Supine MVM | 38 | | 12.48 (9.00) | 35 | 5.89 (6.96) | 4 | .36 | **.00** | |
|  | Standing MVM | 37 | | 6.12 (7.00) | 35 | 6.00 (5.48) | 5 | .07 | .53 | |
| Urethral Mobility | | | | | | | | | | |
| Height of BN (mm) | Supine Cough | | 38 | 12.65 (9.87) | 37 | 9.76 (7.50) | 2 | .51 | **.03** | |
|  | Standing Cough | | 37 | 7.18 (9.13) | 38 | 4.54 (4.10) | 2 | .51 | **.03** | |
| PFM Function | | | | | | | | | | |
| BN Excursion (mm) | Supine MVC | | 38 | 4.37 (3.33) | 37 | 4.05 (4.66) | 2 | .08 | .72 | |
| Height of BN (mm) | Supine MVC | | 38 | 26.80 (7.76) | 38 | 24.21 (7.17) | 1 | .51 | **.03** | |
| LH ML Thickness (mm) | Supine Rest | | 37 | .68 (.18) | 38 | .67 (.13) | 2 | .07 | .54 | |
|  | Standing Rest | | 38 | .77 (.18) | 38 | .75 (.22) | 1 | 0 | .98 | |
|  | Supine MVC | | 35 | .68 (.17) | 34 | .73 (.17) | 8 | .14 | .23 | |
|  | Standing MVC | | 35 | .76 (.12) | 33 | .78 (.17) | 9 | .08 | .54 | |

*Note. Bladder neck (BN); Levator hiatus (LH); Medio-lateral (ML); Levator plate length (LPL); Cross-sectional area (CSA); Maximal voluntary contraction (MVC); Maximal Valsalva maneuver (MVM); Cure is defined as 2g≥ on a pad test.

Appendix 4

Table 3. Group differences in demographic and clinical variables between women with SUI who were cured vs not cured with the physiotherapy intervention.

| Outcome Measure | Mean ± SD | | | | Missing (n) | Cohen’s *d* | *p* |
| --- | --- | --- | --- | --- | --- | --- | --- |
|  | n | Cured | n | Not Cured |  |  |  |
| Age (years) | 37 | 48.65 ± 10.78 | 38 | 51.39 ± 10.11 | 2 | .04 | .26 |
| ICIQ-SF (0-21) | 37 | 11.27 ± 4.64 | 39 | 13.38 ± 3.55 | 1 | .08 | **.03** |
| Baseline Pad Weight | 38 | 11.70 ± 15.37 | 39 | 29.70 ± 20.09 | 0 | .16 | **.00** |
| Outcome | Median (Interquartile Range) | | | | Missing (n) | *r* | *p* |
|  | n | Cured | n | Not Cured |  |  |  |
| BMI (kg/m^2^) | 37 | 27.13 (10.18) | 37 | 25.84 (6.50) | 3 | .28 | .23 |
| Parity | 38 | 2 (2) | 39 | 2 (1) | 0 | .12 | .58 |
| Bodyweight of Heaviest Baby | 31 | 4 (1) | 36 | 4 (1) | 10 | .30 | .21 |
| Number of Vaginal Deliveries | 26 | 2 (1) | 34 | 2 (1) | 17 | .04 | .90 |
| Number of C-Sections | 8 | 1 (1) | 2 | 2 (0) | 67 | .80 | .25 |
| Vacuum Use | 2 | 1 (0) | 4 | 1 (1) | 71 | .61 | .48 |
| Forceps Use | 3 | 1 (0) | 3 | 1 (0) | 71 | 0 | 1.00 |
| PFM Strength | 38 | 4 (1) | 39 | 3 (1) | 0 | .24 | .30 |
| PFM Tone | 38 | 1 (1) | 39 | 0 (1) | 0 | .61 | .01 |

*Note. Short Form International Consultation on Incontinence Questionnaire (ICIQ-SF); Body mass index (BMI); Pelvic floor muscle (PFM); Cure is defined as ≥2g leaked on a pad test.

Appendix 5

Table 4. Group differences in categorical variables between women with SUI who were cured vs not cured with the physiotherapy intervention.

| Categorical Variables | | Frequencies | | Totals | *p* |
| --- | --- | --- | --- | --- | --- |
|  |  | Cured | Not Cured |  |  |
| Smoking | Yes | 5 (6.5%) | 4 (5.2%) | 9 (11.7%) | .69 |
|  | No | 33 (42.9%) | 35 (45.5%) | 68 (88.3%) |  |
| Menopause | Post | 18 (23.4%) | 21 (27.3%) | 39 (50.6%) | .57 |
|  | Pre | 20 (26.0%) | 18 (23.4%) | 38 (49.4%) |  |
| Hormonal Contraceptive Use | Yes | 5 (13.2%) | 3 (7.9%) | 8 (21.1%) | .52 |
|  | No | 15 (39.5%) | 15 (39.5%) | 30 (78.9%) |  |
| Hormonal Replacement Therapy | Yes | 3 (7.7%) | 4 (10.3%) | 7 (17.9%) | .85 |
|  | No | 15 (38.5%) | 17 (43.6%) | 32 (82.1%) |  |
| Hysterectomy | Yes | 5 (6.5%) | 5 (6.5%) | 10 (13.0%) | .97 |
|  | No | 33 (42.9%) | 34 (44.2%) | 67 (87.0%) |  |
| Levator Avulsion | Yes | 2 (2.6%) | 3 (3.9%) | 5 (6.5%) | 1.00 |
|  | No | 36 (46.8%) | 36 (46.8%) | 72 (93.5%) |  |

*Note. Cure is defined as ≥2g urine leakage on a standardized 30-minute pad test.
